# Health and Sport. Economic and Social Impact of Active Tourism

**DOI:** 10.3390/ejihpe10010007

**Published:** 2019-09-05

**Authors:** Noelia Araújo Vila, Jose Antonio Fraiz Brea, Arthur Filipe de Araújo

**Affiliations:** 1 Business and Tourism Faculty, University of Vigo, 32004 Ourense, Spain; jafraiz@uvigo.es; 2 Department of Economics, Management, Industrial Engineering and Tourism, University of Aveiro, 3810193 Aveiro, Portugal; arthuraraujo@ua.pt

**Keywords:** sports, health, active tourism, wellness

## Abstract

Concerns with health and wellness are currently a global trend. Consequently, people have increasingly been engaging in some form of regular sports practice in their adult lives. This is evidenced by the rise of markets that are related to the sports sector. One of those market trends is active tourism, which refers to the practice of physical activities in natural environments within a tourist destination. In this context, people travel to certain places, typically with friends or as couples, to engage in sports activities of greater or lesser intensities. This allows for them to disconnect with their daily routines and provides them all of the positive health-related outcomes that are associated with exercising. Therefore, health tourism is also an efficient tool against sedentariness. The present study explores which sports are currently the most popular amongst active tourists, which attributes they value the most when choosing a destination, and what is their expenditure pattern. To this end, a sample of 60 individuals who have engaged in some form of active tourism in Galicia is surveyed. The findings suggest that active tourists are particularly interested in dedicating their discretionary time to travelling and they have a higher daily expenditure within the destination than the average tourist.

## 1. Introduction

People worldwide are increasingly concerned about health and wellness. Recent improvements in life quality and life expectancy have led people to seek self-realisation in several contexts, such as personal, social, and leisure [1]. In this context, it is more than ever essential to be in a good state of health, to which physical activity and sports play a central role.

A healthy lifestyle, which normally includes practicing some type of sports discipline, is socially developed throughout a person’s life. Therefore, childhood and adolescence are key phases in this process [2]. Throughout a person’s life, physical activities must be adequately practiced, having health and well-being as the main goals [3].

Many studies address the positive impacts of physical activities in the human body [4], especially in the improvement of the cardiovascular system and the consequent prevention of cardiovascular diseases [5,6]. Others focus on sports’ role on people’s mental health and stress management [7]. Another branch of studies addresses the effects of lifelong sports (those that people practice throughout their whole lives) and outdoor activities, which are particularly effective in maintaining health [8]. The habit of practicing outdoor sports, along with recreation and tourism, is taught to very young children as a part of their education. However, sports are beneficial for people of all ages. In the case of tourism, active tourism is an advisable alternative not only for adults, but also for school age children and young people [9]. Active tourism also allows for people to discover new spots and destinations and be in contact with nature. Therefore, it connects sports to vacations, leisure, and fun.

The present work first addresses the health-sports binomial, which is a popular topic in current society. The study then focuses on active tourism, which is a booming tourism sub-sector, also being connected to the social trend of sports and wellness concerns. The first step to addressing those topics was a theoretical review on the influence of sports on human health, as well as on the concept of active tourism and its practices. The empirical element of the study was carried out through a survey with 60 people who have engaged in at least one active tourism activity in Galicia, Spain, a region with a great offer of natural environments and recourses for this type of travel. The goal is to explore the sports activities that arise the most interest among these tourists, as well as the attributes that they consider when choosing a destination and their expenditure patterns. In this context, the study highlights the current relevance of health tourism and the importance that I scurrently given to sports.

## 2. Health and Sports

It is widely acknowledged that sports and physical activities are important both for people’s medical health and subjective well-being. For this reason, they have become social goods [10]. Sports have long been employed as instruments for improving general quality of life [11]. In this context, many European initiatives have been carried out that aim to encourage people to engage in sports activities. Back in 1966, the European Council has implemented the “Sports for all” program, which aimed to foment society’s physical and mental health [12]. In 1975, the European Letter of Sports reinforced the program for All. In 1991 and 2001, the same occurred with the European letter of Sports, in which sports are defined as “all forms of physical activities that, through informal or organised participation, aims to express or improve physical fitness and mental well-being, forming social relationships or obtaining results in all levels of competition” [13].

In this context, an ever-increasing number of individuals regularly practice sports. In the United Kingdom, for instance, 40% of men and women of 16 years of age or more practice sports activities at least once a week. Moreover, sports practice is associated with a reduction of 20% to 40% in mortality by all causes when compared to sedentariness [14]. Previous studies have confirmed that physical activity leads to a reduction on the risk of coronary diseases, obesity, diabetes type 2, and other forms of chronic illnesses [15]. Practicing moderate intensity physical activities during at least 30 min five days a week, or high intensity activities for at least 20 min three days a week, improves one’s functional capacity and reduces the incidence of cardiovascular diseases [16]. Moreover, poor health that is caused by physical inactivity entails high costs with public health. In the UK alone, 1.06 billion pounds per year are spent with treatments, and 35,000 still die due to health problems that are associated with sedentariness [17].

Over time, the understanding of physical activity has evolved, and the guidelines of their practices have been developed accordingly. However, 20% to 40% of the world’s population still lead a sedentary lifestyle [18]. Education on health and physical activity is essential for reducing this figure and improving social welfare. Therefore, institutions that play some role in the health system must seek to improve people’s well-being. One of their main goals is to educate society regarding the concept of health, which is often simply understood as “the absence of disease”. However, since the end of the previous century, health has been viewed as a tendency to achieve an optimal physical, psychological, and social state [19]. Therefore, it is an integrated concept, which extends beyond one’s physical state.

As previously mentioned, sports practice affects people’s health and it must be adequately carried out. In this context, physical activities should be practiced is such a way that they bring about more health benefits than risks. To this end, a set of factors must be considered when practicing sports [20]:

- whether the activity’s intensity might be controlled;
- the safety associated with the environment;
- the abruptness level of the movements involved;
- the risk of impacts: with moving elements or opponents; and,
- psychological and social problems potentially caused by competitiveness, which must be controlled.

Other authors state that regularly practiced moderate physical activity is fundamental to improving life quality, preventing the development or the progression of many chronic diseases, and delaying the effects of age [21]. These activities may be differentiated between recreational and high performance, whereas the former entails lesser risks than the latter [1]. Previous studies [20,22] have established possible benefits of exercise (preventive, rehabilitation, or wellness) and the risks caused by the type of activity, environment, and risk behaviour (Table 1).

Arguably, sports are an intrinsic part of current society. It is part of children’s and adolescents’ learning process during school and arouses the interest of adults, both amateurs and professionals [23]. Historically, the sports considered traditional (football, swimming, boxing, etc.) have been not only widely practiced, but also even more widely consumed by spectators. However, within the last years, sports have started to play another role on society, which is a consequence of the increasing global concern with health. In this context, new disciplines have been created, and old ones, which until very recently were not particularly popular, have received a great amount of attention. The new disciplines include group fitness techniques, which encompass gymnastics and dance, and militarised training, such as CrossFit. The old disciplines that gained extreme popularity include mechanical and routine training in the gym and running [24]. 

In addition to that, people are now willing to travel to practice different sports activities of greater or lesser intensities in their free time, especially in natural environments, which characterises active tourism. This type of travel attracts individuals who dedicate part of their vacation and leisure time to practicing sports activities, either in organised events, through specialised companies or by their own initiative. This fits within the branch of health and wellness practices, combined with sports and the purity of natural environments. 

## 3. Active Tourism

Leisure and tourism activities can be intimately connected [25]. Active tourism emerges from this link. The concept encompasses all kinds of outdoor tourism activities that imply the practice of some physical effort. According to Antar-Ecotono [26] “active tourism is defined as travels motivated by practicing sports activities of different physical intensities, and that make use of natural resources without degrading them”. In this context, it is encompassed by the more comprehensive concept of nature-based tourism [27], which includes a greater set of recreational, interpretation, and educational activities with varying degrees of profoundness, risk, and physical intensity, taking place in natural environments [28]. In this context, active tourism is a relatively recent topic and most of the investigations on it have been published within the last decade [29].

The figures reinforce that this type of tourism is on the rise. 34% of travellers plan to take an active trip, that is, one involving outdoor activities that imply some physical effort (of greater or lesser intensity), in the next 12 months [30]. In 2017, this type of travel grew 40% worldwide as compared to the previous three years.

Regarding the encompassed activities, in the 2000s, Buckle [29] concluded that the sector includes 35 to 40 disciplines: abseiling, acrobatic aircraft flights, ballooning, black water rafting, bungy jumping, caving, cross-country skiing, diving, alpine skiing and snowboarding, expedition cruises, gliding, hang-gliding, heli-skiing and heliboarding, hiking, horse riding, ice climbing, motor boating, kiteboarding, road cycling and mountain biking, mountaineering, driving 4 × 4 vehicles, paragliding, quad biking, rock climbing, kite surfing, sailing, sea kayaking, snowshoeing, surfing, whale watching, canoeing, rafting, wildlife observation, and zorbing.

More recently, the number of encompassed activities has grown, and they are classified according to two criteria: environment (terrestrial, aquatic, and aerial, Table 2) [27] and intensity (soft or hard).

According to Booking.com [30], the most currently sought activities are hiking and cycle tourism, but other types of sport, such as surfing, are also gaining popularity. Terrestrial activities are definitely the most popular, followed more and more closely by aquatic ones.

Active tourism typically takes place in non-overcrowded areas with limited levels of man-made modifications, which gives it a character of purity and tranquillity, also linked to the previously addressed search for well-being. Many studies discuss the benefits of this kind of tourism, which extend beyond physical health. Active tourists are typically environmentally aware, which contributes to the conservation and greater appreciation of the natural environment (as well as to the regeneration of natural spaces). It is a type of tourism that appeals to a very specific, although constantly growing, market. This type of travel directly and indirectly fosters many businesses in the visited places from the economic point of view, especially those that provide the goods and services that are required for the specific activities. In this context, active tourists typically spend more than the average tourist within the destination [31] and, consequently, benefit local populations by generating an additional demand for other types of products and services, such as restaurants [31]. 

## 4. Methodology

The present study’s objective is to verify which outdoor sports are most popular amongst active tourists, which attributes these travellers consider in their choice of destination, and which are the expenditure patterns—and consequently, the economic benefits—of this kind of tourism. To this end, the empirical component of the study took place in Galicia, an autonomous community in the northwest of Spain, known for having many protected natural areas (7th in the Spanish ranking) [32], a big coastline (1498 km), rivers, mountains, and different natural resources that favour the practices of many outdoor sports. The region also includes the destination of the Way of Saint James, as well as a rich cultural heritage and distinct gastronomy, which further enhances its potential. In this context, according to Trivago´s ranking, Galicia is in the top 10 of best active tourism destinations in Spain.

Data were collected through a survey questionnaire, which was divided in three parts. Part I included questions regarding the active tourism activities respondents engaged in; part II encompassed questions about which attributes travellers consider in the active tourism destination choice; part III regards tourists’ expenses during their stay; and, part IV aimed to collect general demographical information about participants. 

The first part consists of two questions, the last active tourism activity practiced (there is a list of several to choose between them) and if you have done any more activity in Galicia. The second part, through a Likert scale of five points (1 being the lowest score and 5 the highest) raises the respondent a series of attributes to assess: accessibility, availability of means of transportation, quality of natural resources, complementary services, signage and information, safety in the activities, cleanliness, and active tourism offer. In the third part, the respondent is asked about the expenses that are incurred in the following activities: accommodation, food, transportation, active tourism activities, complementary tourism activities, and shopping. Finally, they are asked the typical variables of their sociodemographic profile: gender, age, education, main occupation, and monthly family income. Table 3 shows the descriptive analysis of the sociodemographic profile. Each respondent has spent an average time of five minutes to answer said survey, raised in such a way that it did not exceed seven minutes.

For the selection of active tourism activities, Araújo et al.’s [27] list was adopted and updated to current trends (Table 2). The research population consisted of people who had engaged in at least one active tourism activity in Galicia. The questionnaires were operationalised through an online platform, which ensued a random sample of 60 respondents (national tourists). The sample is not large, being the biggest limitation of the study, but lacking a census of active tourists has chosen to contact the active tourism companies of Galicia (a total of 22) and request the dissemination of the link of the survey through their social networks. After several rounds of publication of the survey, it has been the response rate obtained, while considering it sufficient for a first approach to this sector. The survey was collected between February and April 2019.

## 5. Results

Table 3 presents the sample’s demographic profile. Most of the participants were men (61.7%), between 20 and 40 years old. Most had either secondary or higher education, were employed workers, and they had monthly family incomes between 1801 and 2400 euros per month. Moreover, the biggest part of respondents engages in active tourism activities with friends (60%).

Regarding the active tourism activities most recently practiced by participants, hiking, and mountaineering are the most popular—18.3% each, followed by surfing (15%), rafting, (8.3%), mountain biking (6.7%), and paintball (6.7%). Canyoning, canoeing, kayaking, ballooning, climbing, windsurfing, and boat trips all had of 5% or less. Nevertheless, when considering activities previously practiced, hiking is clearly the most popular, as it has been practiced at some point by 44.3% of the sample. Accordingly, rafting (12.5%) figures in second place and kayaking (10.2%), which starts gaining relevance amongst the aquatic activities, in third.

**Figure 1:**
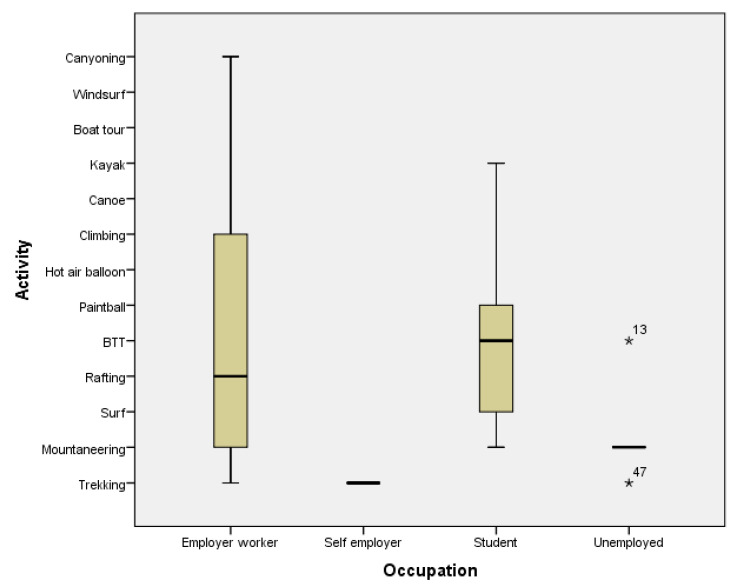
Most recently practiced activity and occupation.

A Pearson’s chi-square test was carried out in order to verify whether the activities practiced were correlated with the demographic variables. As shown in Table 4, the only demographic variable with a significance level lower than 0.05 is occupation. Students are more likely to practice surf, rafting, BTT, and paintball, while unemployed people are more inclined to mountaineering and employed workers prefer more activities (Figure 1). The remaining variables do not show any statistically significant difference.

Eight items were measured through a five-point Likert scale in order to verify which attributes are considered by active tourists in their choice of destination (1: I do not consider it at all; 5: I consider it a lot). The results (Table 5) indicate that the most valued factors are cleanliness, safety and quality of natural resources, all with an average score above 4.25 (out of 5). It should also be observed that all the factors were rated five by at least one respondent, and that cleanliness and safety obtained the highest minimums. Potential correlations between factors have also been analysed. However, the Pearson’s correlation coefficients were not significant (all values are far from 1 and closer to 0, which suggests that there is no correlation between factors).

Possible significant differences correlations between the value given to attributes and sociodemographic variables were also analysed. As shown in Table 6, only formal education and occupation showed values that were lower than 0.05. Regarding formal education, respondents with only secondary education attribute less importance to the availability of means of transportation, which is significantly more valued by participants with higher education. Regarding occupation, safety was significantly valued more by employed workers than it was by self-employed respondents.

To analyse active tourists’ expenses, different cost components were analysed, namely: accommodation, food, transportation, active tourism activities, complementary tourism activities, and shopping. The average individual expense of the inquired active tourists is 162.70 €, and the average stay is 0.95 nights. As shown in Table 7 the highest cost component is food, followed by the active tourism activities *per se* and accommodation. 

Finally, possible significant relationships between tourists’ level of expenditure and sociodemographic profiles have been analysed. In this case, the travel group type has also been considered in the analysis (Table 8). 

Regarding age, respondents that are between 32–36, 48–50, and 52–53 years old spend significantly more with accommodation. When considering travel group type, couples and friend groups show the highest expenses in this category, as well as with food and the active tourism activities *per se*. Expenses with food are higher amongst respondents of 34, 36, and 38 years old, to whom it goes up to 80 € per person. Regarding respondents’ monthly family income, people earning between 1800 and 3000 are the ones who spend the most in the destination. At last, expenses with complementary tourism (tours, day trips, visits to attractions, etc.), higher numbers were found amongst the respondents with only secondary education as well as those earning between 1800 and 3000 euros.

## 6. Conclusions

The value that is currently attributed by society to health and physical appearance has led to an increase in sports practice between adults. Historically, sports have been deemed necessary for children and teenagers and, thus, became an integral part of their education (through the discipline of physical education). Sports have always been though to generate positive effects on children and teenagers; however, people tended to gradually leave them aside as they grew up. Currently, however, sports have become fashionable practices between adults, who engage in different practices in search of health and/or aesthetic pursuits. In this context, new disciplines have emerged or (re)gained popularity, such as CrossFit, Zumba, group dance classes, running, gym training, and outdoor sports activities (e.g., hiking, mountaineering, rafting, etc.). These activities attract each time more people, and even compete with more traditional ones, such as football, basketball, and swimming. This confirms the theories already raised by Bendíková et al. [4] regarding the positive effects of sport. Additionally, individuals and institutions worldwide are increasingly concerned about the consequences of physical inactivity. In this context, governments, and especially public health organisations, make efforts to educate society on the possible consequences of a sedentary lifestyle on one’s health and well-being. In this way, initiatives from the 1960s and 1970s and early 2000s that boosted sport as something healthy for society are resumed [13]. Recent studies have shown that physical inactivity increases the risk of many chronic diseases and health conditions, such as hypertension, diabetes, and obesity.

An increasingly significant part of society is aware of the risks that are brought about by a sedentary lifestyle. This is reflected in the increasing demand in certain health-related markets, such as active tourism [27]. In previous decades, particularly in the end of the XX century, sun and beach tourism (vacations for resting) dominated the industry. Now, there are many new market segments that demand another type of vocation, to which physical activities are an important part, or even the main purpose. Enjoying the beach and sunbathing is no longer enough, as tourists increasingly demand activities and experiences in natural environments that imply some physical effort. In this context, sun and beach tourism is still very popular, however it is increasingly associated with physical activities in coastal areas, such as scuba diving, hiking, and paragliding. De Knop was already advancing this trend in the 1990s [33], so that its continuity and intensification are verified today. 

The same applies to inland areas, which people typically visit in shorter trips (normally one or two nights), during which they seek to enjoy natural landscapes (and thus, escape from their mundane city life), as well as engage in some kind of activity (horse riding, hiking, rafting, canoeing, ballooning, etc.). The intensity of such activities varies, depending on how demanding they are, as well as on each person’s physical form.

The present study shows that sports activities in natural environments increasingly appeal to people of all ages. Moreover, although men are still a majority, each time more women engage in such practices. In addition, although the male gender is still the majority, more and more women are joining this practice, which reduces the gender gap in this sector, something that studies at the end of the last century did affirm [34]. The results also show that this type of tourists typically prefers to travel in couples or with friends. Moreover, active tourists spend more than the average tourist in the destination, as the expenses with the activity *per se* add to those with accommodation, food, and complementary services. In this context, the segment normally attracts people with higher incomes, who value sports activities and are willing to invest in them, confirming previous studies where the necessary equipment for the practice of this type of tourism already increases spending [35]. Although expenses that are directly related to the sports activity are often significant, those with food frequently surpass them, which are also normally higher than those with accommodation in all age groups. At last, it should be observed that this type of tourist normally places a particularly high value on cleanliness in the destination, safety of the activities (and thus, seek experts and renowned companies), and quality of natural resources. Within these three attributes, no respondent scored 1 (I do not consider it at all). I the case of safety in the activities, the lowest score was 3. 

In conclusion, the increase in active tourism demand reflects the social trend of seeking well-being and health through sports practices in natural environments. In this context, an increasing number of people place a high value on these activities and are willing to dedicate part of their discretionary resources to practice them. Even so, it is not mass tourism, part of its charm, being an alternative tourism. In addition, there are large areas where it can be carried out, not involving agglomerations as in other tourist resources. Even so, the offer of this type of tourism is not excessive, with a greater campaign of diffusion and its connection with the sport being necessary, being able to be a practice more of the denominated healthy for the individuals. It is a sport practice that is more linked to tourism and natural environments.

Finally, mention as limitation of the study the sample size, 60 individuals and the origin of these (Spanish). Not having a census of individuals who practice active tourism, they have chosen to contact Galician active tourism companies via social networks and request the dissemination of the survey through their networks. Therefore, the results that were obtained in this study are limited to the Galician territory and national tourists. For future research, we will try to expand the sample (both in size and territory), being able to obtain more consistent results and establish comparisons with other territories.

## Figures and Tables

**Table 1:** Benefits and risks of physical activities.

**Benefits**	**Preventive**	**Rehabilitation**	**Wellness**
	Enhancement of all body systems; prevention of risk factors associated with certain diseases; overweight and obesity control.	Biomedical: faster recovery from accidents, injuries, cardiovascular problems, etc. Psychological: remedy to anxiety, stress and depression. Improved state of mind.	Strong social relationships, general wellness and satisfaction, fun, security, self-esteem, performance, etc.
**Risks**	**Type of activity**	**Environment**	**Risk behaviour**
	Risk of impacts in activities involving abrupt movements and problems caused by competitiveness.	Risks caused by environmental factors (weather, equipment, etc.), falls, accidents related to the natural environment (as in active tourism).	Exercise abuse: injuries, irritability, nervousness, apathy, etc. Misuse: safety issues or inadequateness between activities and personal characteristics.

Source: The authors, adapted from [20,22].

**Table 2:** Classification of activities encompassed by active tourism.

**1. Terrestrial activities**	- Hiking - Mountaineering/mountain climbing - Horse riding - Road cycling and Mountain biking - Speleology - Cycle tourism - Bungee jumping - Paintball - Canyoning	- Cross-country skiing - Badminton - Dog sledge - Snowmobile - Archery - 4 × 4 - Quad biking - Outdoor training - Via ferratas
**2. Aquatic activities**	- Canoeing/kayaking - Fluvial tourism - Diving - Surfing/Windsurfing/Kitesurfing - Nautical motorcycle	- Rafting - Hidrobob/Hidrospeed - Sailing - Water skiing - …
**3. Aereal activities**	- Hang gliding - Hot air balloonning - Paragliding - Slope Skydiving - Skydiging	- Ultralighting - Motorless flying - Heliexcursión - …

Source: [27].

**Table 3:** Sample’s demographic profile.

Variable	Classification	%
Gender	Man	61.7
	Woman	38.7
Age	<=19	3.30
	20–30	28.3
	31–40	30.0
	41–50	21.7
	51–60	16.7
Education	Secondary school	50.0
	Hoigher education	50.0
Main occupation	Employed worker	65.0
	Self-employed	6.7
	Student	16.7
	Unemployed	11.7
Monthly family income	0–600 euros	1.7
	601–1200 euros	3.3
	1201–1800 euros	3.3
	1801–2400 euros	70.0
	2401–3000 euros	15.0
	Más de 3000 euros	6.7
Travel group type	Friends	60.0
	Couple	21.7
	Relatives	18.3

**Table 4:** Pearson’s Chi-square test between the most recently practiced activity and demographic variables.

Variable	Bilateral Asymptote Sig.
Gender	0.694
Age	0.124
Education	0.086
Main occupation	0.01
Monthly family income	0.711

**Table 5:** Evaluation of active tourism destination choice factors.

	N	Minimum	Maximum	Mean	Std. Deviation
**Accessibility**	60	1	5	2.73	1.163
**Availability of means of transportation**	60	1	5	2.38	1.195
**Quality of natural resources**	60	2	5	4.28	0.761
**Complementary services**	60	1	5	3.92	1.013
**Signage and information**	60	1	5	3.27	1.326
**Safety in the activities**	60	3	5	4.38	0.691
**Cleanliness**	60	2	5	4.50	0.701
**Active tourism offer**	60	1	5	3.82	1.172

**Table 6:** Pearson’s Chi-square test between destination choice factors and sociodemographic profiles.

	Gender	Age	Formal Education	Main Occupation	Monthly Family Income
**Accessibility**	0.688	0.204	0.060	0.412	0.627
**Availability of means of transportation**	0.735	0.529	0.017	0.314	0.451
**Quality of natural resources**	0.133	0.431	0.113	0.886	0.188
**Complementary services**	0.932	0.549	0.173	0.331	0.764
**Signage and information**	0.371	0.382	0.607	0.374	0.419
**Safety in the activities**	0.416	0.467	0.237	0.028	0.651
**Cleanliness**	0.858	0.974	0.762	0.194	0.999
**Active tourism offer**	0.213	0.182	0.179	0.460	0.909

**Table 7:** Expense components of active tourists (in euros).

	N	Minimum	Maximum	Mean	Std. Deviation
**Accomodation**	60	0	70	34.67	17.850
**Food**	60	20	80	49.88	17.924
**Transportation**	60	10	35	18.18	6.693
**Active tourism activities**	60	0	80	37.57	23.826
**Complementary tourism activities**	60	0	30	5.42	8.351
**Shopping**	60	0	350	25.72	62.012

**Table 8:** Pearson’s Chi-square test between expense components and sociodemographic profiles.

	Gender	Age	Formal Education	Main Occupation	Monthly Family Income	Travel Group Type
**Accomodation**	0.128	0.035	0.127	0.125	0.292	0.026
**Food**	0.718	0.01	0.234	0.289	0.017	0.003
**Transportation**	0.170	0.367	0.448	0.325	0.787	0.205
**Active tourism activities**	0.331	0.221	0.477	0.241	0.949	0.04
**Complementary tourism activities**	0.458	0.247	0.049	0.888	0.033	0.307
**Shopping**	0.125	0.765	0.628	0.589	0.950	0.331
